# Comparing the Metabolic Capabilities of Bacteria in the *Mycobacterium tuberculosis* Complex

**DOI:** 10.3390/microorganisms7060177

**Published:** 2019-06-18

**Authors:** Rachael A. Fieweger, Kaley M. Wilburn, Brian C. VanderVen

**Affiliations:** Department of Microbiology and Immunology, College of Veterinary Medicine, Cornell University, Ithaca, NY 14850, USA; raf277@cornell.edu (R.A.F.); kmw248@cornell.edu (K.M.W.)

**Keywords:** *Mycobacterium tuberculosis* complex, carbon metabolism, macrophages, tuberculosis, cholesterol, fatty acids

## Abstract

Pathogenic mycobacteria are known for their ability to maintain persistent infections in various mammals. The canonical pathogen in this genus is *Mycobacterium tuberculosis* and this bacterium is particularly successful at surviving and replicating within macrophages. Here, we will highlight the metabolic processes that *M. tuberculosis* employs during infection in macrophages and compare these findings with what is understood for other pathogens in the *M. tuberculosis* complex.

## 1. Introduction

*Mycobacterium tuberculosis* is the causative agent of human tuberculosis (TB) and this bacterium is a member of the closely related cluster of species termed the *Mycobacterium tuberculosis* complex (MTBC). Members of the MTBC cause TB or TB-like disease in humans, livestock, and wildlife with high levels of morbidity and mortality. It is thought that all MTBC pathogens evolved from a common ancestor yet the bacteria fall into phylogenetically distinct lineages and clades under the following species names: *M. tuberculosis*, *M. africanum*, *M. microti*, *M. bovis*, *M. suricattae*, *M. mungi*, *M. orygis*, *M. caprae*, *M. pinnipedii*, and *M. canettii*.

Human-adapted MTBC isolates are categorized into seven distinct lineages. *M. tuberculosis* causes the majority of clinically observed TB cases in humans and is divided into five sub-lineages termed L1-L4 and L7. *M. africanum* causes TB in humans and comprises lineages L5 and L6 [1]. *M. microti* infects voles and occasionally causes TB in immunocompromised humans [2,3]. *M. bovis* can cause TB in humans, wild/domesticated bovines, possums, badgers, cervids, and goats [4]. Humans are considered dead-end hosts for *M. bovis* since human-to-human transmission with *M. bovis* is rarely observed [5]. *M. suricattae* is a meerkat isolate [6,7] and *M. mungi* is a banded mongoose isolate [8]. *M. orygis* has a broad host range including humans [9,10] and *M. caprae* is associated with goats [4]. *M. pinnipedii* is an isolate of seals, sea lions, tapirs, camels, and human animal trainers/workers [11,12,13,14]. Lastly, *M. canettii* is an environmental isolate that causes rare and sporadic, TB-like disease in humans [15,16].

These major lineages in the MTBC are primarily defined by small deletion mutations and these mutations are termed regions of difference or RD [17,18,19,20,21,22]. For example, *M. tuberculosis* H37Rv has 14 regions of difference (RD1–14), ranging in size from 2 to 12 kb, and this genetic material is deleted from the genome of *M. bovis* BCG-Pasteur [20,21,23]. The human-adapted strains, *M. tuberculosis* and *M. africanum*, are differentiated by the RD9 mutation that removed two genes in this region from the *M. africanum* genome [24]. MTBC isolates that rarely infect humans carry the RD9 deletion and lack genetic material within the RD7, RD8, and RD10 regions.

Genome comparisons across the *M. tuberculosis* lineages indicate that specific genes may be under different patterns of selection based on their putative function [25,26,27]. Recent findings indicate that genes associated with metabolism in *M. tuberculosis* are under strong selective pressure in an experimental mouse model of infection [28]. This likely reflects the unique challenges associated with the facultative intracellular lifestyle of MTBC pathogens [29,30,31,32,33,34,35,36,37]. To replicate in macrophages, *M. tuberculosis* employs a specialized metabolism to counter nutrient limitations and the intrinsic immune pressures of macrophages [37]. Features of this specialized metabolism in *M. tuberculosis* are conserved among the MTBC pathogens and this is likely a unifying trait that contributes to the successful colonization of mammalian hosts [38]. Here we will review our current understanding of *M. tuberculosis* metabolism and discuss the metabolic similarities and differences that exist across this important group of pathogens.

## 2. Pathogenic Processes of the Canonical Pathogen, *Mycobacterium tuberculosis*

Human beings are the only natural host and reservoir for *M. tuberculosis*. A defining aspect of this disease is that the bacterium infects many, but causes a debilitating disease in relatively few individuals. In the year 2017 alone, *M. tuberculosis* caused an estimated 10 million new infections and was responsible for 1.6 million deaths [39]. The majority (>90%) of healthy individuals develop asymptomatic TB disease where the immune system contains but may not eliminate the bacteria. Co-morbidities, such as HIV infection that weaken immunity increase the potential for active TB disease, and roughly −10% of immuno-deficient individuals infected with *M. tuberculosis* develop active TB each year [40]. Key to the life cycle of *M. tuberculosis* is the bacterium’s ability to persist and avoid clearance by host immunity between cycles of aerosol transmission.

Aerosol transmission deposits *M. tuberculosis* in the airways, where the bacterium infects alveolar macrophages, monocyte derived macrophages, dendritic cells, and neutrophils [41,42]. These infected cells stimulate expansion and/or recruitment of additional phagocytes and the eventual recruitment of antigen-specific T lymphocytes to the site of infection [43,44,45]. Antigen specific T lymphocytes exert immunologic control over the infection leading to a reduction in bacterial proliferation and limiting dissemination. The chronic nature of the infection promotes tissue damage and formation of granuloma lesions. Typically, TB granulomas are stratified lesions comprised of a necrotic caseous center surrounded by cellular layers enriched in phagocytes and lymphocytes [46]. Within these lesions, *M. tuberculosis* is typically observed in the acellular necrotic debris and/or internalized within phagocytic cells [47,48].

*M. tuberculosis* spends much of its life cycle intracellularly within macrophages and the bacterium avoids elimination within these cells by actively manipulating intracellular trafficking processes [49]. *M. tuberculosis* replicates in macrophage phagosomes do not fully acidify and are maintained at a pH of −6.4 [50,51,52]. These phagosomes maintain vesicular connectivity within the cell, receiving cargo from sorting/recycling endosomes [53] and the secretory pathway [51,54]. In resting macrophages, *M. tuberculosis* accesses nutrients to support bacterial growth within phagosomes [55,56,57,58,59]. Immunologic activation of macrophages with interferon gamma (IFN-γ) disables *M. tuberculosis’s* ability to manipulate intracellular trafficking events, and the bacterial phagosome transforms into a restrictive compartment resembling a hydrolytically active acidified lysosome [60,61]. Activated macrophages do not completely eliminate *M. tuberculosis*, but these cells effectively constrain intracellular replication of the bacteria by limiting nutrient availability, producing nitric oxide and antimicrobial peptides, and autophagy-mediated processes [62,63,64,65]. Thus, even in the context of this single cell type, *M. tuberculosis* is subject to various constraints associated with nutrient availability and immune pressures imposed by the macrophage [64,66].

## 3. The Metabolic Capabilities of *M. tuberculosis*

Some intracellular pathogens are naturally auxotrophic and depend on macrophages to supply particular nutrients [67,68]. In contrast, *M. tuberculosis* is a prototroph and is equipped with the complete catabolic and anabolic pathways required to convert basic organic precursors into most, if not all, essential products (amino acids, co-factors, vitamins, nucleotides) [69]. *M. tuberculosis* also simultaneously metabolizes multiple distinct nutrients *in vitro* [70] and in macrophages [71]. This metabolic flexibility likely allows *M. tuberculosis* to survive under various different nutritional stresses the bacterium encounters across its lifecycle. Therefore, nutrient abundance or availability along with pressures imposed by macrophages likely define some or all metabolic capabilities of *M. tuberculosis* at any given time. Due to the intimate interactions that occur between *M. tuberculosis* and the macrophage, we will focus on the bacterium’s metabolism in this cell type and discuss themes that span across MTBC human and animal pathogens.

## 4. Carbon Metabolism

It has long been understood that *M. tuberculosis* preferentially utilizes host fatty acids *in vivo* [72]. Studies conducted over the past twenty years have all concluded that fatty acids are important carbon sources for *M. tuberculosis* during infection in macrophages [56,57,66,73,74,75]. More recently, it has become clear that cholesterol plays an equally important role in *M. tuberculosis* metabolism during macrophage infection [57,64]. *M. tuberculosis* uses fatty acids and cholesterol to fuel aneplurotic and energy producing reactions of central metabolism. Additionally, these substrates are used to synthesize bacterial lipid virulence factors that antagonize the immune response, promote pathogenicity, and facilitate persistence [76].

Bacterial degradation of cholesterol is unusual and relatively few bacteria are known to metabolize this molecule [77,78]. Saprophytic Mycobacteria subsist on dead or decaying organic matter in the environment and the ability to metabolize phytosterols or cholesterol from plant matter or animal carcasses likely provides a growth advantage in the environment [79]. The cholesterol metabolic pathway is conserved across the MTBC, and a recent genome comparison study predicted that a functional pathway is present in 51 of the 93 mycobacterial species examined [80]. Cholesterol is a mammalian cell membrane lipid, which macrophages acquires through endocytosis [81] and sequesters following inflammation [82]. It is likely that the ability to metabolize cholesterol provided an evolutionary advantage allowing an ancient MTBC bacteria, permitting colonization and survival in macrophages of mammals.

**Figure 1:**
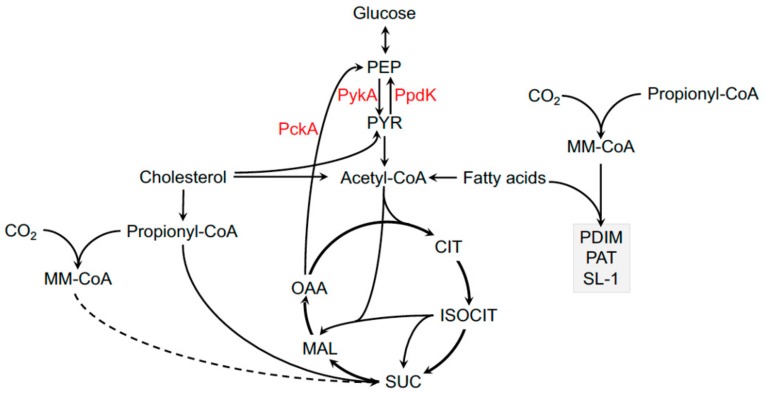
Central metabolic pathways of *Mycobacterium tuberculosis*. Central metabolic enzymes discussed in text indicated in red and methyl-branched polyketide lipids indicated in grey box. PEP, phosphoenolpyruvate; PYR, pyruvate; CIT, citrate; ISOCIT, isocitrate; SUC, succinate; MAL, malate; OOA, oxaloacetate; MM-CoA, methylmalonyl-CoA; PDIM, phthiocerol-dimycocerosate; PAT, polyacyl-trehalose; SL-1, sulfolipid; PckA, phosphoenolpyruvate carboxykinase; PykA, pyruvate kinase; PpdK, pyruvate phosphate dikinase.

*M. tuberculosis* can metabolize carbohydrates *in vitro*, but the bacterium utilizes these carbohydrates to sustain homeostasis and/or biosynthetic reactions rather than energy production [83,84]. During infection, *M. tuberculosis* primarily synthesizes carbohydrates through the gluconeogenic conversion of lipid- and/or amino acid-derived intermediates [85]. Inactivating the gluconeogenic enzyme PckA in *M. bovis* BCG or *M. tuberculosis* restricts bacterial growth in macrophages [86,87]. Other bacteria in the MTBC likely synthesize carbohydrates via gluconeogenesis. MTBC isolates that rarely infect humans and strains and the human adapted *M. africanum* lineages L5 and L6 have a dead-end glycolysis pathway due to an E200D substitution in the pyruvate kinase enzyme, PykA [1,88]. PykA catalyzes the final step in glycolysis, and this mutation prevents the bacteria from growing on carbohydrate substrates. This mutation explains why pyruvate rather than glycerol has classically been needed to culture certain animal adapted isolates in the lab (Figure 1) [89].

MTBC bacteria also have increased flexibility around the pyruvate node in metabolism and can convert pyruvate into phosphoenolpyruvate (PEP), which may be important when the bacteria metabolize cholesterol. Cholesterol degradation produces excess pyruvate and this intermediate can fuel gluconeogenesis through the production of PEP via pyruvate phosphate dikinase, PpdK activity (Figure 1) [90]. While subtle differences exist across the MTBC, the overwhelming evidence indicates that all of these bacteria primarily synthesize carbohydrates via gluconeogenesis and preferentially utilize lipids and/or amino acids to fuel central metabolic pathways.

## 5. Lipid Import

*M. tuberculosis* uses Mce transporters to import lipid substrates across the bacterial cell wall [76]. The *M. tuberculosis* genome encodes four separate Mce transporters termed Mce1-4 and it is known that Mce4 imports cholesterol [57,64] while Mce1 imports fatty acids [57,73]. These transporters are encoded individual operons within the *M. tuberculosis* genome and have long been implicated in the virulence of *M. tuberculosis*, particularly to promote bacterial growth in macrophages [91]. *M. tuberculosis* mutants lacking Mce4 replicate poorly in IFN-γ activated macrophages and have a slow growth phenotype in chronic infected mice [64]. It is unclear if this slow growth phenotype is due to restricted bacterial access to cholesterol, or an increased demand by the bacterium for cholesterol under conditions of IFN-γ activation. Expression of the Mce4 transporter varies across *M. tuberculosis* and is highly expressed by isolates from lineages L1 and L6 during infection in macrophages [92]. Proteins of the Mce1 transporter have long been considered virulence factors and are highly conserved across members of the MTBC [91].

The genes encoding the Mce1 transporter are considered core genes in the MTBC bacteria [93,94]. Informatic predictions suggest that *M. africanum* isolates in lineages L5 and L6 may carry a mutation in the *Mce1B* subunit but it is unknown if this mutation impacts Mce1 transporter activity [95]. Horizontal gene transfer is known to occur between environmental mycobacteria [96]. This process is thought to have been widespread in saprophytes prior to speciation in mammals [97]. Environmental mycobacteria commonly transfer genes to metabolize: 1) Amino acids and derivatives; 2) carbohydrates; 3) cofactors, vitamins, prosthetic groups, and pigments; and 4) lipids [98]. Interestingly, *M. chubuense*, a non-pathogenic environmental isolate, carries a plasmid with an intact Mce1 operon embedded in a transposon, suggesting that the ability to import fatty acids can be mobilized between mycobacterial species in the environment [98].

The substrates of the Mce2 and Mce3 transporters in *M. tuberculosis* remain unknown, but it is likely that these protein complexes are also transporters of hydrophobic nutrients. In the context of MTBC evolution, it is noteworthy to indicate that MTBC isolates that rarely infect humans and *M. africanum* lineage L6 lack the Mce3 transporter due to an eight-gene deletion associated with the RD7 mutation [19]. It is likely that this RD7 mutation prevents the import and metabolism of a yet-unknown hydrophobic substrate that these pathogens do not require. Defining the substrate imported by Mce3 in *M. tuberculosis* will shed light on one major metabolic difference across the MTBC.

## 6. Fatty Acid Metabolism in *M. tuberculosis*

Fatty acids are a versatile carbon source for *M. tuberculosis*. The bacteria can -oxidize fatty acids to fuel gluconeogenesis and energy-producing central metabolic pathways or *M. tuberculosis* can use fatty acids for biosynthesis. *M. tuberculosis* shunts fatty acids to polyketide synthases for elongation into methyl-branched virulence lipids [99] or mycobactin to scavenge iron [100]. Fatty acids can also be elongated to form cell wall mycolic acids, or incorporated into membrane phospholipids [101]. Furthermore, fatty acids can be converted into triacylglycerol, which is thought to function as a carbon reserve, which can be metabolized when nutrients are limiting [102,103]. Similarly, *M. bovis* incorporates fatty acids as biosynthetic precursors to produce lipid-based end-products [104,105,106,107,108]. While the fate of fatty acids in the metabolism of animal-adapted MTBC remains poorly characterized it is expected that these processes are highly conserved across the MTBC members given the closely related lipid end-products these bacteria produce.

## 7. Cholesterol Metabolism in *M. tuberculosis*

Unlike fatty acids, it appears that cholesterol is degraded by *M. tuberculosis* exclusively to release acetyl-CoA, propionyl-CoA, and pyruvate all of which, fuel central metabolic pathways in the bacterium [76]. It is now appreciated that this cocktail of metabolic intermediates lies at a critical axis in *M. tuberculosis* metabolism that fuels energy-producing metabolism, gluconeogenesis, and biosynthetic pathways (Figure 1). The cholesterol-derived intermediate, propionyl-CoA, feeds into central metabolism via the methyl-citrate cycle (MCC) [109,110] or the methyl-malonyl pathway (MMP) [111]. Propionyl-CoA is also used to synthesize methyl-branched polyketide lipids (Figure 1) [99]. The unique flexibility of propionyl-CoA in *M. tuberculosis* metabolism allows the bacteria to sustain central metabolic pathways and balance toxic effects associated with cholesterol utilization (see below) [76,112,113].

The MCC assimilates propionyl-CoA into central metabolism via succinate and pyruvate, which occurs in a vitamin B12 (B12)-independent manner. In contrast, the MMP assimilates propionyl-CoA as succinyl-CoA in a B12-dependent manner (Figure 1). Bacteria and archaea generally synthesize B12, whereas humans and animals absorb most of this essential vitamin from their diet [114]. *M. tuberculosis* is not known to produce B12 *in vitro* or in macrophages suggesting the bacteria assimilates B12 or a B12 precursor directly from the host [115]. The soil dwelling bacteria, *M. canettii*, has every gene required to synthesize B12 while human- and animal-adapted isolates in the MTBC lack the B12 biosynthetic enzyme, CobF [116]. Thus, the inability to fully synthesize B12 may represent a specific patho-adaptation by members of the MTBC that reflects an increased dependence on B12 obtained from mammalian hosts.

Cholesterol degradation produces excess propionyl-CoA, which induce a metabolic toxicity in *M. tuberculosis* if the bacterium is unable to properly assimilate this intermediate [56,113]. Shunting propionyl-CoA into the biosynthesis of lipids can minimize the toxicity [56]. Through the biosynthesis of methyl-branched lipids, propionyl-CoA is assimilated as methyl-branched subunits in phthiocerol-dimycocerosate (PDIM), polyacylated trehalose (PAT), and sulfolipid (SL) [99]. During infection, *M. tuberculosis* produces excess PDIM and SL as a result of coupling cholesterol metabolism to the synthesis of these lipids [117,118,119]. It is currently unknown if this metabolic coupling links the production of virulence factors to an environmental condition or if this phenomenon simply reflects the excess amount of cholesterol that is metabolized by *M. tuberculosis* during infection.

PDIM has been implicated in macrophage invasion and recruitment [120,121], resistance to immune mediated stress [122,123,124], masking cell wall antigens [121], and facilitating bacterial escape from macrophage phagosomes [125,126]. All of these activities could be influenced by increased production of PDIM and/or other cell wall polyketide virulence lipids. Analysis of bacterial gene expression of MTBC lineages found that *M. africanum* isolates from L6 represses genes associated with PDIM synthesis during infection in macrophages [92]. The majority of human- and animal-adapted MTBC strains produce a modified variant of PDIM that contains a phenolic glycolipid moiety, while most laboratory and European strains of *M. tuberculosis* do not produce this modified PDIM due to a mutation in pks15/1 [127]. The glycosylation patterns on this lipid vary across the MTBC [128], and this feature has been proposed to alter pathogenesis [129] and/or the immune response to this lipid [130,131].

## 8. Coupled Metabolism of Fatty Acids and Cholesterol in the Macrophage

Recent studies have revealed a “codependency” of fatty acids and cholesterol in *M. tuberculosis* metabolism. As mentioned above, cholesterol metabolism is associated with a metabolic toxicity and *M. tuberculosis* mitigates this by synthesizing methyl-branched polyketide lipids. Importantly the bacterium’s ability to synthesize methyl-branched lipids is limited by the amount of available fatty acid primers. For example, cholesterol-mediated toxicity in an *M. tuberculosis* mutant lacking Icl1 is reversed by supplying excess fatty acids to infected macrophages and these fatty acids become incorporated into PDIM [56]. Thus, *M. tuberculosis* likely co-metabolizes fatty acids and cholesterol such that fatty acids are available as a “sink” for excess propionyl-CoA assimilation in the form of methyl-branched lipids such as PDIM. Thus, an important aspect of *M. tuberculosis* metabolism is to balance fatty acid and cholesterol utilization during infection.

## 9. *M. tuberculosis* Metabolic Flexibility and Macrophage Heterogeneity

Macrophages develop from distinct cell lineages and evidence is accumulating suggesting that developmental origin influences how these cells ultimately respond to infection and injury [132,133]. In mice, *M. tuberculosis* primarily resides in bone marrow derived, interstitial macrophages and, within alveolar macrophages that arise from the fetal liver during embryonic development [134]. The interstitial macrophages display an M1-like activation phenotype while the alveolar macrophages display an M2-like activation phenotype [134]. Additionally, in non-human primates, *M. tuberculosis* also resides in macrophages that express the M1 and M2 activation markers [135].

Importantly, these murine macrophage subsets have highly polarized metabolic states, where the M1-like macrophages are actively glycolytic and the M2-like macrophages are actively undergoing fatty acid oxidation [134]. This suggests that the types, and amounts of nutrients available to *M. tuberculosis* during infection vary with the host cell and the flexible metabolism of the bacterium may allow adaptation and survival within different macrophage cell types [134]. It is possible that in M2-like alveolar macrophages, *M. tuberculosis* may benefit from the increased import of lipids by the macrophage [134]. Perhaps in M1-like interstitial macrophages, *M. tuberculosis* may preferentially metabolize glycolysis end products such as lactate that accumulate in these cells [37,136]. It is currently unclear how *M. tuberculosis* fuels energy producing pathways in M1-like macrophages but *ex vivo* studies with human monocyte derived macrophages have demonstrated that *M. tuberculosis* can sustain a gluconeogenic pathway by assimilating lactate [137]. Going forward it will be interesting to understand how the metabolism of MTBC pathogens differs in specific host cells.

## 10. Nitrogen from Amino Acid Metabolism in *M. tuberculosis*

All cells require nitrogen to synthesize amino acids, nucleotides, and various essential cofactors. Recent studies have begun to shed light on how *M. tuberculosis* assimilates and metabolizes nitrogen during infection in macrophages. When cultured *in vitro*, *M. tuberculosis* demonstrates a preference for amino acids as a source of nitrogen over ammonia chloride, and *M. tuberculosis* also has the ability to co-metabolize multiple amino acids simultaneously [138]. During infection in macrophages it is thought that *M. tuberculosis* encounters nitrogen in two forms: As a nitrate derived from NO and as individual amino acids. In activated macrophages, *M. tuberculosis* can reduce nitrate (NO_3−_) to nitrite (NO_2−_) through the activity of the multi-subunit nitrate reductase, NarGHJI [139,140]. By using nitrate as an electron acceptor the bacterium can maintain respiration or cellular homeostasis when oxygen is not available or limiting [141]. *M. tuberculosis* can also assimilate nitrogen from amino acids and when infected macrophages are pulsed with ^15^N-labeled amino acids, both aspartate and asparagine accumulate in the bacterial phagosome and the bacteria require these amino acids to maintain infections [142,143]. Additionally, metabolic tracing experiments in macrophages labeled with ^13^C-glucose established that a portion of the ^13^C-label accumulates in host cell amino acids and when these cells are infected *M. tuberculosis* is able to scavenge the^13^C-labeled amino acids [71].

Nitrate reductase activity has been used for over 50 years in diagnostic mycobacteriology to distinguish *M. tuberculosis* from animal-adapted members of the MTBC. *M. tuberculosis* possesses high levels of nitrate reductase activity relative to MTBC isolates that rarely infect humans, which corresponds to promoter mutations upstream of the nitrate reductase operon in these isolates [144]. A recent gene expression study comparing the five distinct MTBC lineages found that nitrate reductase is highly expressed in lineages L2 and L4 but is repressed in lineages L1 and L6 during infection in macrophages [92]. This finding is consistent with previous observations that clinical isolates from lineage L1 are variable with respect to their nitrate reductase phenotype [145]. In *M. tuberculosis*, nitrate reductase activity has been associated with mitigating acid and reactive nitrogen stresses under hypoxic conditions [146], however it is unclear if nitrate reductase activity confers a specific fitness advantage in different MTBC lineages. The mycobacterial nitrate reductase likely requires molybdenum cofactor (MoCo) for enzymatic activity [147,148]. Interestingly, MTBC isolates in clade A3 lack many genes thought be involved in MoCo biosynthesis (see below), suggesting that a specific patho-adaptation has occurred in this clade that may further reduce nitrate reductase activity in isolates from clade A3.

## 11. Metals and Metabolism in *M. tuberculosis*

*M. tuberculosis* requires metals such as iron and copper for growth, and these elements are linked to growth and pathogenicity of *M. tuberculosis* in macrophages [149]. Iron is required for the activity of numerous enzymes, as it is either attached in a heme nucleus or coordinated by amino acid side chains within enzyme active sites. During infection in macrophages *M. tuberculosis* induces the expression of many genes associated with acquiring and sequestering iron [74,75,150]. *M. tuberculosis* acquires iron via siderophore scavenging from iron-loaded transferrin in the endocytic pathway [51,53] or by importing heme [151]. Metal composition analysis by X-ray fluorescence in infected macrophages found that *M. tuberculosis* containing phagosomes have high concentrations of iron, a property that was dependent on the production of siderophores and did not occur in nonpathogenic mycobacteria [152]. Similarly, copper plays an important role in *M. tuberculosis* metabolism because it forms the nuclear center of the *aa*_3_-type cytochrome *c* oxidase [141,153]. This cytochrome oxidase is one of two enzymes in *M. tuberculosis* that are known to require copper for activity and this cytochrome oxidase is critical for oxidative respiration. Copper also accumulates in the phagosomes of infected macrophages, which presumably provides the bacterium necessary levels of this metal [152]. While it is not clear how copper is imported by *M. tuberculosis*, it appears that excess copper is associated with adverse effects on *M. tuberculosis*, and the bacterium has several ways to remove or inactivate this metal to avoid toxicity (see below).

## 12. Molybdenum Cofactor in *M. tuberculosis*

MoCo is a cyclic redox cofactor required by a subset of enzymes to catalyze carbon, nitrogen, and sulfur metabolic reactions across all kingdoms of life [154]. The *M. tuberculosis* genome encodes numerous putative MoCo requiring enzymes, including the nitrate reductase NarGHJI [155] along with an expanded set of genes thought to be involved in MoCo biosynthesis [156]. Although the role of MoCo in *M. tuberculosis* metabolism during infection remains unknown, the expansion of genes involved in MoCo is a unique feature of MTBC pathogens [157]. The biosynthesis of MoCo in *M. tuberculosis* is a complicated and poorly-understood process that likely involves multiple redundant enzymes [156]. A subset of these MoCo biosynthetic genes (*moaA1-D1*) was likely acquired by *M. tuberculosis* during an ancient horizontal gene transfer event [158,159,160] and these genes enhance survival of *M. tuberculosis* during hypoxia [147]. Interestingly, *M. orygis* isolates in the clade A3 carry a unique 13 gene deletion mutation (RD12oryx) that disables multiple putative MoCo biosynthetic genes and a transcriptional regulator of the *moaA1-D1* operon [147,161]. It is unknown if these bacteria produce MoCo but, it is tempting to speculate that a unique patho-adaptation has occurred in these isolates that impacts the ability of these bacteria to produce MoCo and tolerate hypoxia.

## 13. Immunological Pressures on *M. tuberculosis* Metabolism

Macrophages are not a static niche that simply supplies nutrients for *M. tuberculosis* and immunologic pressures imposed by macrophages modulate the bacterium’s ability to engage metabolic pathways and utilize host-derived nutrients. Immune activation induces expression of the macrophage immunoresponsive gene-1 (Irg1 or aconitate decarboxylase 1) [162]. This enzyme disables the macrophage mitochondrial TCA cycle by decarboxylating cis-aconitate [162] leading to the accumulation of acetyl-CoA, which can be diverted into fatty acid synthesis by the host cell. Additionally, the cis-aconitate decarboxylation reaction also produces itaconic acid and this metabolite inhibits both *M. tuberculosis* and macrophage metabolism. Itaconic acid inhibits Icl1 in *M. tuberculosis*, which limits the bacterium’s ability to utilize intermediates from cholesterol and fatty acid degradation [109]. Itaconic acid also regulates mitochondrial respiration by inhibiting succinate dehydrogenase, leading to the production of excess succinate that drives glycolytic metabolism in the macrophage and modulation of the immune response [163]. Amino acids are also a heavily contested nutrient during infection. IFN-γ-induced activation of macrophages increases expression of the enzyme indoleamine 2, 3-dioxygenase 1 (Ido1) [164]. The host protein Ido1 degrades tryptophan and depletes levels of this critical amino acid in activated cells; however, *M. tuberculosis* compensates for this by synthesizing its own tryptophan [165,166]. Similarly, mice infected with *M. bovis* increases overexpression of Ido1 although this bacterium also synthesizes its own tryptophan [167]. Given that activation of Ido-1 is produced in response to the cell mediated adaptive immunity it is expected that all members of the MTBC will experience this constraint and rely on the synthesis of this amino acid to replicate during infection.

Macrophages also possess an armamentarium of metal-based mechanisms to eliminate intracellular pathogens [168]. Although copper and zinc are important metals in *M. tuberculosis* physiology these metals also constitute an important cellular defense when these ions accumulate within macrophage lysosomes during infection [168]. The P-type ATPase ATP7A mediates phagosomal accumulation of copper, which functions as a defense mechanism to intoxicate bacterial pathogens [169]. To mitigate copper overload and toxicity *M. tuberculosis* either actively secretes copper [170,171], or sequesters the metal in an inert, protein-bound form [172]. Macrophages are thought to deliver zinc into phagosomes through the activity of one or more transporters [173] and *M. tuberculosis* mitigates zinc poisoning by actively exporting the metal [174]. The understanding that *M. tuberculosis* is equipped to mitigate copper and zinc toxicity during infection in macrophages is a relative recent finding. It will be exciting to determine how other MTBC members adapt to these metals during infection.

## 14. Acidic pH and PhoPR

*M. tuberculosis* senses and adapts to the environmental conditions within macrophage phagosomes [48] and one major signal sensed by *M. tuberculosis* is fluctuating acidity [150]. *M. tuberculosis* senses and adapts to low pH environments via the two-component regulator system, PhoPR [175]. The PhoPR system is critical for virulence/pathogenesis that controls the expression of genes in a large virulence regulon involved in metabolism [176], virulence protein secretion (Esx) [177,178], and the biosynthesis of methyl-branched lipids (acylated trehalose, SL, PDIM) [175,179].

Most human-adapted isolates of the MTBC have a functional PhoPR system while *M. africanum* and MTBC isolates that rarely infect humans have a mutation in the sensor domain of the PhoR kinase (PhoR G71I), which disables this protein [180]. It is well established that many genes in the PhoPR regulon are required for virulence/pathogenesis. Bacteria that carry a non-functional PhoR also have compensatory mutations that partly restore expression of the PhoPR regulon [181]. Specifically, some lineages with a non-functional PhoR have a secondary deletion termed RD8. The RD8 mutation restores the expression and secretion of virulence proteins (Esx) in *M. africanum* lineage L6 and related isolates ^181^. Isolates in *M. africanum* lineage L5 also carry a non-functional PhoR but these bacteria do not have a RD8 mutation. *M. africanum* lineage L5 bacteria are still able to express and secrete virulence proteins (Esx) indicating that an unidentified mechanism compensates for the PhoR mutation in this lineage [182].

Methyl-branched lipid biosynthesis is also regulated by the PhoPR system and these lipids have long been associated with virulence in *M. tuberculosis* [183]. Additionally, synthesis of these lipids can mitigate metabolic toxicity associated with cholesterol and fatty acid metabolism (see above) [56]. It is tempting to speculate that low pH-dependent activation of methyl-branched lipid synthesis via PhoPR activation could couple host-derived lipid utilization pathways in *M. tuberculosis* to promote pathogenesis in macrophages.

These methyl-branched polyketide lipids may also play a role in transmission. Specifically, *M. bovis* isolates rarely transmit between humans, with a notable exception being the *M. bovis* isolate strain B. This strain of *M. bovis* efficiently transmits between humans and has been associated with high mortality among HIV-infected persons [184]. Molecular characterization of this strain revealed an IS*6110* insertion upstream of *phoP* that enhances transcription of *phoP* [185]. Importantly, when this mutation was engineered into a laboratory isolate of *M. bovis* this mutation allowed the bacteria to produce SL, a methyl-branched polyketide lipid [180]. This observation suggests that the virulence and/or host range of MTBC bacteria can be altered by the PhoPR-dependent production of methyl-branched polyketide lipids such as SL.

## 15. Concluding Remarks

Differences clearly exist between the MTBC members with respect to pathogenesis, host range, and transmission there are also similarities with these pathogens. This review focused on the specialized metabolism that allows *M. tuberculosis* to survive in macrophages because this is a common survival strategy used by MTBC pathogens that rarely infects humans. Comparative genomic approaches across members of the MTBC revealed several key differences regarding virulence and immunogenicity. Similarly, we are optimistic that comparative approaches focused on metabolism will enhance our understanding of this important group of pathogens and the animals they infect.

## References

[1] De Jong B.C., Antonio M., Gagneux S. (2010). *Mycobacterium africanum*—Review of an important cause of human tuberculosis in West Africa. PLoS Negl. Trop. Dis..

[2] Niemann S., Richter E., Dalügge-Tamm H., Schlesinger H., Graupner D., Königstein B., Gurath G., Greinert U., Rüsch-Gerdes S. (2000). Two cases of *Mycobacterium microti* derived tuberculosis in HIV-negative immunocompetent patients. Emerg. Infect. Dis..

[3] Van Soolingen D., van der Zanden A.G., de Haas P.E., Noordhoek G.T., Kiers A., Foudraine N.A., Portaels F., Kolk A.H., Kremer K., van Embden J.D. (1998). Diagnosis of *Mycobacterium microti* infections among humans by using novel genetic markers. J. Clin. Microbiol..

[4] Malone K.M., Gordon S.V., Gagneux S. (2017). Strain Variation in the Mycobacterium tuberculosis Complex.: Its Role in Biology, Epidemiology and Control.

[5] Blazquez J., Espinosa de Los Monteros L.E., Samper S., Martín C., Guerrero A., Cobo J., Van Embden J., Baquero F., Gómez-Mampaso E. (1997). Genetic characterization of multidrug-resistant *Mycobacterium bovis* strains from a hospital outbreak involving human immunodeficiency virus-positive patients. J. Clin. Microbiol..

[6] Dippenaar A., Dippenaar A., Parsons S.D.C., Sampson S.L., van der Merwe R.G., Drewe J.A., Abdallah A.M., Siame K.K., Gey van Pittius N.C., van Helden P.D. (2015). Whole genome sequence analysis of *Mycobacterium suricattae*. Tuberculosis.

[7] Parsons S.D., Drewe J.A., Gey van Pittius N.C., Warren R.M., van Helden P.D. (2013). Novel cause of tuberculosis in meerkats, South Africa. Emerg. Infect. Dis..

[8] Alexander K.A., Laver P.N., Michel A.L., Williams M., van Helden P.D., Warren R.M., Gey van Pittius N.C. (2010). Novel *Mycobacterium tuberculosis* complex pathogen, *M. mungi*. Emerg. Infect. Dis..

[9] Marcos L.A., Spitzer E.D., Mahapatra R., Ma Y., Halse T.A., Shea J., Isabelle M., Lapierre P., Escuyer V.E. (2017). *Mycobacterium orygis* Lymphadenitis in New York, USA. Emerg. Infect. Dis..

[10] Rahim Z., Thapa J., Fukushima Y., van der Zanden A.G.M., Gordon S.V., Suzuki Y., Nakajima C. (2017). Tuberculosis Caused by *Mycobacterium orygis* in Dairy Cattle and Captured Monkeys in Bangladesh: A New Scenario of Tuberculosis in South Asia. Transbound. Emerg. Dis..

[11] Cousins D.V., Bastida R., Cataldi A., Quse V., Redrobe S., Dow S., Duignan P., Murray A., Dupont C., Ahmed N. (2003). Tuberculosis in seals caused by a novel member of the *Mycobacterium tuberculosis* complex: *Mycobacterium pinnipedii* sp. nov. Int. J. Syst. Evol. Microbiol..

[12] Forshaw D., Phelps G.R. (1991). Tuberculosis in a captive colony of pinnipeds. J. Wildl. Dis..

[13] Moser I., Prodinger W.M., Hotzel H., Greenwald R., Lyashchenko K.P., Bakker D., Gomis D., Seidler T., Ellenberger C., Hetzel U. (2008). *Mycobacterium pinnipedii*: Transmission from South American sea lion (*Otaria byronia*) to Bactrian camel (*Camelus bactrianus bactrianus*) and Malayan tapirs (*Tapirus indicus*). Vet. Microbiol..

[14] Thompson P.J., Cousins D.V., Gow B.L., Collins D.M., Williamson B.H., Dagnia H.T. (1993). Seals, seal trainers, and mycobacterial infection. Am. Rev. Respir Dis..

[15] Pfyffer G.E., Auckenthaler R., van Embden J.D., van Soolingen D. (1998). *Mycobacterium canettii*, the smooth variant of *M. tuberculosis*, isolated from a swiss patient exposed in Africa. Emerg. Infect. Dis..

[16] Van Soolingen D., Hoogenboezem T., de Haas P.E., Hermans P.W., Koedam M.A., Teppema K.S., Brennan P.J., Besra G.S., Portaels F., Top J. (1997). A novel pathogenic taxon of the *Mycobacterium tuberculosis* complex, canetti: Characterization of an exceptional isolate from Africa. Int. J. Syst. Bacteriol..

[17] Behr M.A., Wilson M.A., Gill W.P., Salamon H., Schoolnik G.K., Rane S., Small P.M. (1999). Comparative genomics of BCG vaccines by whole-genome DNA microarray. Science.

[18] Brosch R., Gordon S.V., Billault A., Garnier T., Eiglmeier K., Soravito C., Barrell B.G., Cole S.T. (1998). Use of a *Mycobacterium tuberculosis* H37Rv bacterial artificial chromosome library for genome mapping, sequencing, and comparative genomics. Infect. Immun..

[19] Brosch R., Gordon S.V., Marmiesse M., Brodin P., Buchrieser C., Eiglmeier K., Garnier T., Gutierrez C., Hewinson G., Kremer K. (2002). A new evolutionary scenario for the *Mycobacterium tuberculosis* complex. PNAS.

[20] Gordon S.V., Brosch R., Billault A., Garnier T., Eiglmeier K., Cole S.T. (1999). Identification of variable regions in the genomes of *tubercle bacilli* using bacterial artificial chromosome arrays. Mol. Microbiol..

[21] Mahairas G.G., Sabo P.J., Hickey M.J., Singh D.C., Stover C.K. (1996). Molecular analysis of genetic differences between *Mycobacterium bovis* BCG and virulent *M. bovis*. J. Bacteriol..

[22] Mostowy S., Tsolaki A.G., Small P.M., Behr M.A. (2003). The *in vitro* evolution of BCG vaccines. Vaccine.

[23] Brosch R., Philipp W.J., Stavropoulos E., Colston M.J., Cole S.T., Gordon S.V. (1999). Genomic analysis reveals variation between *Mycobacterium tuberculosis* H37Rv and the attenuated *M. tuberculosis* H37Ra strain. Infect. Immun..

[24] Mostowy S., Onipede A., Gagneux S., Niemann S., Kremer K., Desmond E.P., Kato-Maeda M., Behr M. (2004). Genomic analysis distinguishes *Mycobacterium africanum*. J. Clin. Microbiol..

[25] Comas I., Chakravartti J., Small P.M., Galagan J., Niemann S., Kremer K., Ernst J.D., Gagneux S. (2010). Human T cell epitopes of *Mycobacterium tuberculosis* are evolutionarily hyperconserved. Nat. Genet..

[26] Coscolla M., Copin R., Sutherland J., Gehre F., de Jong B., Owolabi O., Mbayo G., Giardina F., Ernst J.D., Gagneux S. (2015). *M. tuberculosis* T Cell Epitope Analysis Reveals Paucity of Antigenic Variation and Identifies Rare Variable TB Antigens. Cell Host Microbe.

[27] Pepperell C.S., Casto A.M., Kitchen A., Granka J.M., Cornejo O.E., Holmes E.C., Birren B., Galagan J., Feldman M.W. (2013). The role of selection in shaping diversity of natural *M. tuberculosis* populations. PLoS Pathog..

[28] Copin R., Wang X., Louie E., Escuyer V., Coscolla M., Gagneux S., Palmer G.H., Ernst J.D. (2016). Within Host Evolution Selects for a Dominant Genotype of *Mycobacterium tuberculosis* while T Cells Increase Pathogen Genetic Diversity. PLoS Pathog..

[29] Alexander K.A., Laver P.N., Williams M.C., Sanderson C.E., Kanipe C., Palmer M.V. (2018). Pathology of the Emerging *Mycobacterium tuberculosis* Complex Pathogen, *Mycobacterium mungi*, in the Banded Mongoose (Mungos mungo). Vet. Pathol..

[30] Amato B., Capucchio T.M., Biasibetti E., Mangano E., Boniotti B.M., Pacciarini L.M., Migliore S., Vitale M., Fiasconaro M., Di Marco Lo Presti V. (2017). Pathology and genetic findings in a rare case of mycobacterium caprae infection in a sow. Vet. Microbiol..

[31] Bouzid F., Brégeon F., Lepidi H., Donoghue H.D., Minnikin D.E., Drancourt M. (2017). Ready Experimental Translocation of *Mycobacterium canettii* Yields Pulmonary Tuberculosis. Infect. Immun..

[32] Coscolla M., Lewin A., Metzger S., Maetz-Rennsing K., Calvignac-Spencer S., Nitsche A., Dabrowski P.W., Radonic A., Niemann S., Parkhill J. (2013). Novel *Mycobacterium tuberculosis* complex isolate from a wild chimpanzee. Emerg. Infect. Dis..

[33] Drewe J.A., Foote A.K., Sutcliffe R.L., Pearce G.P. (2009). Pathology of *Mycobacterium bovis* infection in wild meerkats (*Suricata suricatta*). J. Comp. Pathol..

[34] Kipar A., Burthe S.J., Hetzel U., Rokia M.A., Telfer S., Lambin X., Birtles R.J., Begon M., Bennett M. (2014). *Mycobacterium microti tuberculosis* in its maintenance host, the field vole (*Microtus agrestis*): Characterization of the disease and possible routes of transmission. Vet. Pathol..

[35] Pollock J.M., Neill S.D. (2002). *Mycobacterium bovis* infection and tuberculosis in cattle. Vet. J..

[36] Roe W.D., Lenting B., Kokosinska A., Hunter S., Duignan P.J., Gartrell B., Rogers L., Collins D.M., de Lisle G.W., Gedye K. (2019). Pathology and molecular epidemiology of *Mycobacterium pinnipedii tuberculosis* in native New Zealand marine mammals. PLoS ONE.

[37] Russell D.G., Huang L., VanderVen B.C. (2019). Immunometabolism at the interface between macrophages and pathogens. Nat. Rev. Immunol..

[38] Yruela I., Contreras-Moreira B., Magalhaes C., Osorio N.S., Gonzalo-Asensio J. (2016). *Mycobacterium tuberculosis* Complex Exhibits Lineage-Specific Variations Affecting Protein Ductility and Epitope Recognition. Genome Biol. Evol..

[39] WHO Global Tuberculosis Report 2018. https://www.who.int/tb/publications/global_report/en/.

[40] Pai M., Behr M.A., Dowdy D., Dheda K., Divangahi M., Boehme C.C., Ginsberg A., Swaminathan S., Spigelman M., Getahun H. (2016). Tuberculosis. Nat. Rev. Dis. Primers.

[41] Cohen S.B., Gern B.H., Delahaye J.L., Adams K.N., Plumlee C.R., Winkler J.K., Sherman D.R., Gerner M.Y., Urdahl K.B. (2018). Alveolar Macrophages Provide an Early *Mycobacterium tuberculosis* Niche and Initiate Dissemination. Cell Host Microbe.

[42] Russell D.G. (2013). The evolutionary pressures that have molded *Mycobacterium tuberculosis* into an infectious adjuvant. Curr. Opin. Microbiol..

[43] Domingo-Gonzalez R., Prince O., Cooper A., Khader S.A. (2016). Cytokines and Chemokines in *Mycobacterium tuberculosis* Infection. Microbiol. Spectr..

[44] Norris B.A., Ernst J.D. (2018). Mononuclear cell dynamics in *M. tuberculosis* infection provide opportunities for therapeutic intervention. PLoS Pathog..

[45] O’Garra A., Redford P.S., McNab F.W., Bloom C.I., Wilkinson R.J., Berry M.P. (2013). The immune response in *tuberculosis*. Annu. Rev. Immunol..

[46] Martin C.J., Carey A.F., Fortune S.M. (2016). A bug’s life in the granuloma. Semin. Immunopathol..

[47] McClean C.M., Tobin D.M. (2016). Macrophage form, function, and phenotype in mycobacterial infection: Lessons from tuberculosis and other diseases. Pathog. Dis..

[48] Russell D.G., VanderVen B.C., Lee W., Abramovitch R.B., Kim M.J., Homolka S., Niemann S., Rohde K.H. (2010). *Mycobacterium tuberculosis* Wears What It Eats. Cell Host Microbe.

[49] Stutz M.D., Clark M.P., Doerflinger M., Pellegrini M. (2018). *Mycobacterium tuberculosis*: Rewiring host cell signaling to promote infection. J. Leukoc. Biol..

[50] Russell D.G., Dant J., Sturgill-Koszycki S. (1996). *Mycobacterium avium*- and *Mycobacterium tuberculosis*-containing vacuoles are dynamic, fusion-competent vesicles that are accessible to glycosphingolipids from the host cell plasmalemma. J. Immunol..

[51] Sturgill-Koszycki S., Schaible U.E., Russell D.G. (1996). Mycobacterium-containing phagosomes are accessible to early endosomes and reflect a transitional state in normal phagosome biogenesis. Embo J..

[52] Pethe K., Swenson D.L., Alonso S., Anderson J., Wang C., Russell D.G. (2004). Isolation of *Mycobacterium tuberculosis* mutants defective in the arrest of phagosome maturation. Proc. Natl. Acad. Sci. USA.

[53] Clemens D.L., Horwitz M.A. (1996). The *Mycobacterium tuberculosis* phagosome interacts with early endosomes and is accessible to exogenously administered transferrin. J. Exp Med..

[54] Fratti R.A., Chua J., Deretic V. (2002). Cellubrevin alterations and *Mycobacterium tuberculosis* phagosome maturation arrest. J. Biol. Chem..

[55] Gouzy A., Poquet Y., Neyrolles O. (2014). Amino acid capture and utilization within the *Mycobacterium tuberculosis* phagosome. Future Microbiol..

[56] Lee W., VanderVen B.C., Fahey R.J., Russell D.G. (2013). Intracellular *Mycobacterium tuberculosis* exploits host-derived fatty acids to limit metabolic stress. J. Biol. Chem..

[57] Nazarova E.V., Montague C.R., La T., Wilburn K.M., Sukumar N., Lee W., Caldwell S., Russell D.G., VanderVen B.C. (2017). Rv3723/LucA coordinates fatty acid and cholesterol uptake in *Mycobacterium tuberculosis*. eLife.

[58] Nazarova E.V., Podinovskaia M., Russell D.G., VanderVen B.C. (2018). Flow Cytometric Quantification of Fatty Acid Uptake by *Mycobacterium tuberculosis* in Macrophages. Bio-Protocol.

[59] Neyrolles O., Wolschendorf F., Mitra A., Niederweis M. (2015). Mycobacteria, metals, and the macrophage. Immunol. Rev..

[60] Schaible U.E., Sturgill-Koszycki S., Schlesinger P.H., Russell D.G. (1998). Cytokine activation leads to acidification and increases maturation of *Mycobacterium avium*-containing phagosomes in murine macrophages. J. Immunol..

[61] Via L.E., Fratti R.A., McFalone M., Pagan-Ramos E., Deretic D., Deretic V. (1998). Effects of cytokines on mycobacterial phagosome maturation. J. Cell Sci..

[62] Alonso S., Pethe K., Russell D.G., Purdy G.E. (2007). Lysosomal killing of mycobacterium mediated by ubiquitin-derived peptides is enhanced by autophagy. Proc. Natl. Acad. Sci. USA.

[63] MacMicking J.D., North R.J., LaCourse R., Mudgett J.S., Shah S.K., Nathan C.F. (1997). Identification of nitric oxide synthase as a protective locus against tuberculosis. Proc. Natl. Acad. Sci. USA.

[64] Pandey A.K., Sassetti C.M. (2008). Mycobacterial persistence requires the utilization of host cholesterol. Proc. Natl. Acad. Sci. USA.

[65] Upadhyay S., Mittal E., Philips J.A. (2018). Tuberculosis and the art of macrophage manipulation. Pathog. Dis..

[66] McKinney J.D., Höner zu Bentrup K., Muñoz-Elías E.J., Miczak A., Chen B., Chan W.T., Swenson D., Sacchettini J.C., Jacobs W.R.J., Russell D.G. (2000). Persistence of *Mycobacterium tuberculosis* in macrophages and mice requires the glyoxylate shunt enzyme isocitrate lyase. Nature.

[67] Alkhuder K., Meibom K.L., Dubail I., Dupuis M., Charbit A. (2009). Glutathione provides a source of cysteine essential for intracellular multiplication of *Francisella tularensis*. PLoS Pathog..

[68] George J.R., Pine L., Reeves M.W., Harrell W.K. (1980). Amino acid requirements of *Legionella pneumophila*. J. Clin. Microbiol..

[69] Cole S.T., Brosch R., Parkhill J., Garnier T., Churcher C., Harris D., Gordon S.V., Eiglmeier K., Gas S., Barry C.E. (1998). Deciphering the biology of *Mycobacterium tuberculosis* from the complete genome sequence. Nature.

[70] De Carvalho L.P., Fischer S.M., Marrero J., Nathan C., Ehrt S., Rhee K.Y. (2010). Metabolomics of *Mycobacterium tuberculosis* reveals compartmentalized co-catabolism of carbon substrates. Chem. Biol..

[71] Beste D.J., Nöh K., Niedenführ S., Mendum T.A., Hawkins N.D., Ward J.L., Beale M.H., Wiechert W., McFadden J. (2013). 13C-flux spectral analysis of host-pathogen metabolism reveals a mixed diet for intracellular *Mycobacterium tuberculosis*. Chem. Biol..

[72] Bloch H., Segal W. (1956). Biochemical differentiation of Mycobacterium tuberculosis grown in vivo and in vitro. J. Bacteriol..

[73] Nazarova E.V., Montague C.R., Huang L., La T., Russell D., VanderVen B.C. (2019). The genetic requirements of fatty acid import by *Mycobacterium tuberculosis* within macrophages. eLife.

[74] Rohde K.H., Veiga D.F., Caldwell S., Balazsi G., Russell D.G. (2012). Linking the transcriptional profiles and the physiological states of *Mycobacterium tuberculosis* during an extended intracellular infection. PLoS Pathog..

[75] Schnappinger D., Ehrt S., Voskuil M.I., Liu Y., Mangan J.A., Monahan I.M., Dolganov G., Efron B., Butcher P.D., Nathan C. (2003). Transcriptional adaptation of *Mycobacterium tuberculosis* within macrophages: Insights into the phagosomal environment. J. Exp. Med..

[76] Wilburn K.M., Fieweger R.A., VanderVen B.C. (2018). Cholesterol and fatty acids grease the wheels of *Mycobacterium tuberculosis* pathogenesis. Pathog. Dis..

[77] Wipperman M.F., Sampson N.S., Thomas S.T. (2014). Pathogen roid rage: Cholesterol utilization by *Mycobacterium tuberculosis*. Crit. Rev. Biochem. Mol. Biol..

[78] Yam K.C., Okamoto S., Roberts J.N., Eltis L.D. (2011). Adventures in rhodococcus - from steroids to explosives. Can. J. Microbiol..

[79] Bergstrand L.H., Cardenas E., Holert J., Van Hamme J.D., Mohn W.W. (2016). Delineation of Steroid-Degrading Microorganisms through Comparative Genomic Analysis. mBio.

[80] Van Wyk R., van Wyk M., Mashele S.S., Nelson D.R., Syed K. (2019). Comprehensive Comparative Analysis of Cholesterol Catabolic Genes/Proteins in Mycobacterial Species. Int. J. Mol. Sci..

[81] Maxfield F.R. (2014). Role of endosomes and lysosomes in human disease. Cold Spring Harbor Perspect. Biol..

[82] Remmerie A., Scott C.L. (2018). Macrophages and lipid metabolism. Cell. Immunol..

[83] Marrero J., Trujillo C., Rhee K.Y., Ehrt S. (2013). Glucose phosphorylation is required for *Mycobacterium tuberculosis* persistence in mice. PLoS Pathog..

[84] Trujillo C., Blumenthal A., Marrero J., Rhee K.Y., Schnappinger D., Ehrt S. (2014). Triosephosphate isomerase is dispensable in vitro yet essential for *Mycobacterium tuberculosis* to establish infection. mBio.

[85] Rhee K.Y., de Carvalho L.P., Bryk R., Ehrt S., Marrero J., Park S.W., Schnappinger D., Venugopal A., Nathan C. (2011). Central carbon metabolism in *Mycobacterium tuberculosis*: An unexpected frontier. Trends Microbiol..

[86] Liu K., Yu J., Russell D.G. (2003). pckA-deficient Mycobacterium bovis BCG shows attenuated virulence in mice and in macrophages. Microbiology.

[87] Marrero J., Rhee K.Y., Schnappinger D., Pethe K., Ehrt S. (2010). Gluconeogenic carbon flow of tricarboxylic acid cycle intermediates is critical for *Mycobacterium tuberculosis* to establish and maintain infection. Proc. Natl. Acad. Sci. USA.

[88] Garnier T., Eiglmeier K., Camus J.C., Medina N., Mansoor H., Pryor M., Duthoy S., Grondin S., Lacroix C., Monsempe C. (2003). The complete genome sequence of *Mycobacterium bovis*. PNAS.

[89] Keating L.A., Wheeler P.R., Mansoor H., Inwald J.K., Dale J., Hewinson R.G., Gordon S.V. (2005). The pyruvate requirement of some members of the *Mycobacterium tuberculosis* complex is due to an inactive pyruvate kinase: Implications for *in vivo* growth. Mol. Microbiol..

[90] Griffin J.E., Gawronski J.D., Dejesus M.A., Ioerger T.R., Akerley B.J., Sassetti C.M. (2011). High-Resolution Phenotypic Profiling Defines Genes Essential for Mycobacterial Growth and Cholesterol Catabolism. PLoS Pathog..

[91] Zhang F., Xie J.P. (2011). Mammalian cell entry gene family of Mycobacterium tuberculosis. Mol. Cell. Biochem..

[92] Homolka S., Niemann S., Russell D.G., Rohde K.H. (2010). Functional genetic diversity among *Mycobacterium tuberculosis* complex clinical isolates: Delineation of conserved core and lineage-specific transcriptomes during intracellular survival. PLoS Pathog..

[93] Casali N., Riley L.W. (2007). A phylogenomic analysis of the Actinomycetales mce operons. BMC Genom..

[94] Marmiesse M., Brodin P., Buchrieser C., Gutierrez C., Simoes N., Vincent V., Glaser P., Cole S.T., Brosch R. (2004). Macro-array and bioinformatic analyses reveal mycobacterial ‘core’ genes, variation in the ESAT-6 gene family and new phylogenetic markers for the *Mycobacterium tuberculosis* complex. Microbiology.

[95] Winglee K., Manson McGuire A., Maiga M., Abeel T., Shea T., Desjardins C.A., Diarra B., Baya B., Sanogo M., Diallo S. (2016). Whole Genome Sequencing of *Mycobacterium africanum* Strains from Mali Provides Insights into the Mechanisms of Geographic Restriction. PLoS Negl. Trop. Dis..

[96] Boritsch E.C., Khanna V., Pawlik A., Honoré N., Navas V.H., Ma L., Bouchier C., Seemann T., Supply P., Stinear T.P. (2016). Key experimental evidence of chromosomal DNA transfer among selected tuberculosis-causing mycobacteria. Proc. Natl. Acad. Sci. USA.

[97] Behr M.A. (2013). Evolution of Mycobacterium tuberculosis. Adv. Exp. Med. Biol..

[98] Das S., Pettersson B.M., Behra P.R., Ramesh M., Dasgupta S., Bhattacharya A., Kirsebom L.A. (2015). Characterization of Three *Mycobacterium* spp. with Potential Use in Bioremediation by Genome Sequencing and Comparative Genomics. Genome Biol. Evol..

[99] Quadri L.E. (2014). Biosynthesis of mycobacterial lipids by polyketide synthases and beyond. Crit. Rev. Biochem. Mol. Biol..

[100] Krithika R., Marathe U., Saxena P., Ansari M.Z., Mohanty D., Gokhale R.S. (2006). A genetic locus required for iron acquisition in *Mycobacterium tuberculosis*. Proc. Natl. Acad. Sci. USA.

[101] Marrakchi H., Laneelle M.A., Daffe M. (2014). Mycolic acids: Structures, biosynthesis, and beyond. Chem. Biol..

[102] Daniel J., Deb C., Dubey V.S., Sirakova T.D., Abomoelak B., Morbidoni H.R., Kolattukudy P.E. (2004). Induction of a novel class of diacylglycerol acyltransferases and triacylglycerol accumulation in *Mycobacterium tuberculosis* as it goes into a dormancy-like state in culture. J. Bacteriol..

[103] Daniel J., Maamar H., Deb C., Sirakova T.D., Kolattukudy P.E. (2011). *Mycobacterium tuberculosis* uses host triacylglycerol to accumulate lipid droplets and acquires a dormancy-like phenotype in lipid-loaded macrophages. PLoS Pathog..

[104] Fernandes N.D., Kolattukudy P.E. (1996). Cloning, sequencing and characterization of a fatty acid synthase-encoding gene from *Mycobacterium tuberculosis* var. *bovis* BCG. Gene.

[105] Gobin J., Wong D.K., Gibson B.W., Horwitz M.A. (1999). Characterization of exochelins of the *Mycobacterium bovis* type strain and BCG substrains. Infect. Immun..

[106] Kikuchi S., Rainwater D.L., Kolattukudy P.E. (1992). Purification and characterization of an unusually large fatty acid synthase from *Mycobacterium tuberculosis* var. bovis BCG. Arch. Biochem. Biophys..

[107] Rainwater D.L., Kolattukudy P.E. (1983). Synthesis of mycocerosic acids from methylmalonyl coenzyme A by cell-free extracts of *Mycobacterium tuberculosis* var. bovis BCG. J. Biol. Chem..

[108] Vergnolle O., Xu H., Blanchard J.S. (2013). Mechanism and regulation of mycobactin fatty acyl-AMP ligase FadD33. J. Biol. Chem..

[109] Eoh H., Rhee K.Y. (2014). Methylcitrate cycle defines the bactericidal essentiality of isocitrate lyase for survival of Mycobacterium tuberculosis on fatty acids. Proc. Natl. Acad. Sci. USA.

[110] Muñoz-Elías E.J., Upton A.M., Cherian J., McKinney J.D. (2006). Role of the methylcitrate cycle in *Mycobacterium tuberculosis* metabolism, intracellular growth, and virulence. Mol. Microbiol..

[111] Savvi S., Warner D.F., Kana B.D., McKinney J.D., Mizrahi V., Dawes S.S. (2008). Functional Characterization of a Vitamin B12-Dependent Methylmalonyl Pathway in *Mycobacterium tuberculosis*: Implications for Propionate Metabolism during Growth on Fatty Acids. J. Bacteriol..

[112] Horswill A.R., Escalante-Semerena J.C. (2001). *In vitro* conversion of propionate to pyruvate by Salmonella enterica enzymes: 2-methylcitrate dehydratase (PrpD) and aconitase Enzymes catalyze the conversion of 2-methylcitrate to 2-methylisocitrate. Biochemistry.

[113] Upton A.M., McKinney J.D. (2007). Role of the methylcitrate cycle in propionate metabolism and detoxification in *Mycobacterium smegmatis*. Microbiology.

[114] Kozyraki R., Cases O. (2013). Vitamin B12 absorption: Mammalian physiology and acquired and inherited disorders. Biochimie.

[115] Gopinath K., Venclovas Č., Ioerger T.R., Sacchettini J.C., McKinney J.D., Mizrahi V., Warner D.F. (2013). A vitamin B(1)(2) transporter in *Mycobacterium tuberculosis*. Open Biol..

[116] Boritsch E.C., Supply P., Honoré N., Seemann T., Stinear T.P., Brosch R. (2014). A glimpse into the past and predictions for the future: The molecular evolution of the tuberculosis agent. Mol. Microbiol..

[117] Griffin J.E., Pandey A.K., Gilmore S.A., Mizrahi V., McKinney J.D., Bertozzi C.R., Sassetti C.M. (2012). Cholesterol catabolism by *Mycobacterium tuberculosis* requires transcriptional and metabolic adaptations. Chem. Biol..

[118] Jain M., Petzold C.J., Schelle M.W., Leavell M.D., Mougous J.D., Bertozzi C.R., Leary J.A., Cox J.S. (2007). Lipidomics reveals control of *Mycobacterium tuberculosis* virulence lipids via metabolic coupling. Proc. Natl. Acad. Sci. USA.

[119] Yang X., Nesbitt N.M., Dubnau E., Smith I., Sampson N.S. (2009). Cholesterol Metabolism Increases the Metabolic Pool of Propionate in *Mycobacterium tuberculosis*. Biochemistry.

[120] Astarie-Dequeker C., Le Guyader L., Malaga W., Seaphanh F.K., Chalut C., Lopez A., Guilhot C. (2009). Phthiocerol dimycocerosates of *M. tuberculosis* participate in macrophage invasion by inducing changes in the organization of plasma membrane lipids. PLoS Pathog..

[121] Cambier C.J., Takaki K.K., Larson R.P., Hernandez R.E., Tobin D.M., Urdahl K.B., Cosma C.L., Ramakrishnan L. (2014). Mycobacteria manipulate macrophage recruitment through coordinated use of membrane lipids. Nature.

[122] Day T.A., Mittler J.E., Nixon M.R., Thompson C., Miner M.D., Hickey M.J., Liao R.P., Pang J.M., Shayakhmetov D.M., Sherman D.R. (2014). *Mycobacterium tuberculosis* strains lacking surface lipid phthiocerol dimycocerosate are susceptible to killing by an early innate host response. Infect. Immun..

[123] Kirksey M.A., Tischler A.D., Siméone R., Hisert K.B., Uplekar S., Guilhot C., McKinney J.D. (2011). Spontaneous phthiocerol dimycocerosate-deficient variants of *Mycobacterium tuberculosis* are susceptible to gamma interferon-mediated immunity. Infect. Immun..

[124] Rousseau C., Winter N., Pivert E., Bordat Y., Neyrolles O., Avé P., Huerre M., Gicquel B., Jackson M. (2004). Production of phthiocerol dimycocerosates protects *Mycobacterium tuberculosis* from the cidal activity of reactive nitrogen intermediates produced by macrophages and modulates the early immune response to infection. Cell. Microbiol..

[125] Barczak A.K., Avraham R., Singh S., Luo S.S. (2017). Systematic, multiparametric analysis of *Mycobacterium tuberculosis* intracellular infection offers insight into coordinated virulence. PLos Pathog..

[126] Quigley J., Hughitt V.K., Velikovsky C.A., Mariuzza R.A., El-Sayed N.M. (2017). The Cell Wall Lipid PDIM Contributes to Phagosomal Escape and Host Cell Exit of *Mycobacterium tuberculosis*. mBio.

[127] Constant P., Perez E., Malaga W., Lanéelle M.A., Saurel O., Daffé M., Guilhot C. (2002). Role of the pks15/1 Gene in the Biosynthesis of Phenolglycolipids in the *Mycobacterium tuberculosis* Complex. J. Biol. Chem..

[128] Malaga W., Constant P., Euphrasie D., Cataldi A., Daffé M., Reyrat J.M., Guilhot C. (2008). Deciphering the genetic bases of the structural diversity of phenolic glycolipids in strains of the *Mycobacterium tuberculosis* complex. J. Biol. Chem..

[129] Reed M.B., Domenech P., Manca C., Su H., Barczak A.K., Kreiswirth B.N., Kaplan G., Barry C.E. (2004). A glycolipid of hypervirulent tuberculosis strains that inhibits the innate immune response. Nature.

[130] Cambier C.J., O’Leary S.M., O’Sullivan M.P., Keane J., Ramakrishnan L. (2017). Phenolic Glycolipid Facilitates Mycobacterial Escape from Microbicidal Tissue-Resident Macrophages. Immunity.

[131] Manca C., Reed M.B., Freeman S., Mathema B., Kreiswirth B., Barry C.E., Kaplan G. (2004). Differential monocyte activation underlies strain-specific *Mycobacterium tuberculosis* pathogenesis. Infect. Immun..

[132] Ginhoux F., Guilliams M. (2016). Tissue-Resident Macrophage Ontogeny and Homeostasis. Immunuity.

[133] Ginhoux F., Jung S. (2014). Monocytes and macrophages: Developmental pathways and tissue homeostasis. Nat. Rev. Immunol..

[134] Huang L., Nazarova E.V., Tan S., Liu Y., Russell D.G. (2018). Growth of *Mycobacterium tuberculosis in vivo* segregates with host macrophage metabolism and ontogeny. J. Exp. Med..

[135] Mattila J.T., Ojo O.O., Kepka-Lenhart D., Marino S., Kim J.H., Eum S.Y., Via L.E., Barry C.E., Klein E., Kirschner D.E. (2013). Microenvironments in tuberculous granulomas are delineated by distinct populations of macrophage subsets and expression of nitric oxide synthase and arginase isoforms. J. Immunol..

[136] O’Neill L.A., Pearce E.J. (2016). Immunometabolism governs dendritic cell and macrophage function. J. Exp. Med..

[137] Billig S., Schneefeld M., Huber C., Grassl G.A., Eisenreich W., Bange F.C. (2017). Lactate oxidation facilitates growth of *Mycobacterium tuberculosis* in human macrophages. Sci. Rep..

[138] Agapova A., Serafini A., Petridis M., Hunt D.M., Garza-Garcia A., Sohaskey C.D., de Carvalho L.P.S. (2019). Flexible nitrogen utilisation by the metabolic generalist pathogen *Mycobacterium tuberculosis*. eLife.

[139] Cunningham-Bussel A., Zhang T., Nathan C.F. (2013). Nitrite produced by *Mycobacterium tuberculosis* in human macrophages in physiologic oxygen impacts bacterial ATP consumption and gene expression. Proc. Natl. Acad. Sci. USA.

[140] Jung J.Y., Madan-Lala R., Georgieva M., Rengarajan J., Sohaskey C.D., Bange F.C., Robinson C.M. (2013). The intracellular environment of human macrophages that produce nitric oxide promotes growth of mycobacteria. Infect. Immun..

[141] Cook G.M., Hards K., Vilcheze C., Hartman T., Berney M. (2014). Energetics of Respiration and Oxidative Phosphorylation in Mycobacteria. Microbiol. Spectr..

[142] Gouzy A., Larrouy-Maumus G., Bottai D., Levillain F., Dumas A., Wallach J.B., Caire-Brandli I., de Chastellier C., Wu T.D., Poincloux R. (2014). *Mycobacterium tuberculosis* exploits asparagine to assimilate nitrogen and resist acid stress during infection. PLoS Pathog..

[143] Gouzy A. (2013). *Mycobacterium tuberculosis* nitrogen assimilation and host colonization require aspartate. Nat. Chem. Biol..

[144] Stermann M., Bohrssen A., Diephaus C., Maass S., Bange F.C. (2003). Polymorphic nucleotide within the promoter of nitrate reductase (NarGHJI) is specific for *Mycobacterium tuberculosis*. J. Clin. Microbiol..

[145] Goh K.S., Rastogi N., Berchel M., Huard R.C., Sola C. (2005). Molecular evolutionary history of tubercle bacilli assessed by study of the polymorphic nucleotide within the nitrate reductase (narGHJI) operon promoter. J. Clin. Microbiol..

[146] Tan M.P., Sequeira P., Lin W.W., Phong W.Y., Cliff P., Ng S.H., Lee B.H., Camacho L., Schnappinger D., Ehrt S. (2010). Nitrate respiration protects hypoxic *Mycobacterium tuberculosis* against acid- and reactive nitrogen species stresses. PLoS ONE.

[147] Levillain F., Poquet Y., Mallet L., Mazères S., Marceau M., Brosch R., Bange F.C., Supply P., Magalon A., Neyrolles O. (2017). Horizontal acquisition of a hypoxia-responsive molybdenum cofactor biosynthesis pathway contributed to *Mycobacterium tuberculosis* pathoadaptation. PLoS Pathog..

[148] Williams M.J., Shanley C.A., Zilavy A., Peixoto B., Manca C., Kaplan G., Orme I.M., Mizrahi V., Kana B.D. (2015). bis-Molybdopterin guanine dinucleotide is required for persistence of *Mycobacterium tuberculosis* in guinea pigs. Infect. Immun..

[149] Fang Z., Sampson S.L., Warren R.M., Gey van Pittius N.C., Newton-Foot M. (2015). Iron acquisition strategies in mycobacteria. Tuberculosis.

[150] Rohde K.H., Abramovitch R.B., Russell D.G. (2007). *Mycobacterium tuberculosis* invasion of macrophages: Linking bacterial gene expression to environmental cues. Cell Host Microbe.

[151] Tullius M.V., Harmston C.A., Owens C.P., Chim N., Morse R.P., McMath L.M., Iniguez A., Kimmey J.M., Sawaya M.R., Whitelegge J.P. (2011). Discovery and characterization of a unique mycobacterial heme acquisition system. Proc. Natl. Acad. Sci. USA.

[152] Wagner D., Maser J., Lai B., Cai Z., Barry C.E., Höner Zu Bentrup K., Russell D.G., Bermudez L.E. (2005). Elemental analysis of *Mycobacterium avium*-, *Mycobacterium tuberculosis*-, and *Mycobacterium smegmatis*-containing phagosomes indicates pathogen-induced microenvironments within the host cell’s endosomal system. J. Immunol..

[153] Megehee J.A., Hosler J.P., Lundrigan M.D. (2006). Evidence for a cytochrome bcc-aa3 interaction in the respiratory chain of *Mycobacterium smegmatis*. Microbiology.

[154] Mendel R.R. (2013). The molybdenum cofactor. J. Biol. Chem..

[155] Williams M.J., Kana B.D., Mizrahi V. (2011). Functional analysis of molybdopterin biosynthesis in mycobacteria identifies a fused molybdopterin synthase in *Mycobacterium tuberculosis*. J. Bacteriol..

[156] Williams M., Mizrahi V., Kana B.D. (2014). Molybdenum cofactor: A key component *of Mycobacterium tuberculosis* pathogenesis?. Crit. Rev. Microbiol..

[157] McGuire A.M., Weiner B., Park S.T., Wapinski I., Raman S., Dolganov G., Peterson M., Riley R., Zucker J., Abeel T. (2012). Comparative analysis of mycobacterium and related actinomycetes yields insight into the evolution of *Mycobacterium tuberculosis* pathogenesis. BMC Genom..

[158] Becq J., Gutierrez M.C., Rosas-Magallanes V., Rauzier J., Gicquel B., Neyrolles O., Deschavanne P. (2007). Contribution of horizontally acquired genomic islands to the evolution of the *Tubercle bacilli*. Mol. Biol. Evol..

[159] Stinear T.P., Seemann T., Harrison P.F., Jenkin G.A., Davies J.K., Johnson P.D., Abdellah Z., Arrowsmith C., Chillingworth T., Churcher C. (2008). Insights from the complete genome sequence of *Mycobacterium marinum* on the evolution of *Mycobacterium tuberculosis*. Genome Res..

[160] Veyrier F., Pletzer D., Turenne C., Behr M.A. (2009). Phylogenetic detection of horizontal gene transfer during the step-wise genesis of *Mycobacterium tuberculosis*. BMC Evol. Biol..

[161] Van Ingen J., Rahim Z., Mulder A., Boeree M.J., Simeone R., Brosch R., van Soolingen D. (2012). Characterization of mycobacterium orygis as *M. tuberculosis* complex subspecies. Emerg. Infect. Dis..

[162] Michelucci A., Cordes T., Ghelfi J., Pailot A., Reiling N., Goldmann O., Binz T., Wegner A., Tallam A., Rausell A. (2013). Immune-responsive gene 1 protein links metabolism to immunity by catalyzing itaconic acid production. Proc. Natl. Acad. Sci. USA.

[163] Lampropoulou V., Sergushichev A., Bambouskova M., Nair S., Vincent E.E., Loginicheva E., Cervantes-Barragan L., Ma X., Huang S.C., Griss T. (2016). Itaconate Links Inhibition of Succinate Dehydrogenase with Macrophage Metabolic Remodeling and Regulation of Inflammation. Cell Metab..

[164] Murray P.J. (2016). Amino acid auxotrophy as a system of immunological control nodes. Nat. Immunol.

[165] Blumenthal A., Nagalingam G., Huch J.H., Walker L., Guillemin G.J., Smythe G.A., Ehrt S., Britton W.J., Saunders B.M. (2012). *M. tuberculosis* induces potent activation of IDO-1, but this is not essential for the immunological control of infection. PLoS ONE.

[166] Zhang Y.J., Reddy M.C., Ioerger T.R., Rothchild A.C., Dartois V., Schuster B.M., Trauner A., Wallis D., Galaviz S., Huttenhower C. (2013). Tryptophan biosynthesis protects mycobacteria from CD4 T-cell-mediated killing. Cell.

[167] Moreau M., Lestage J., Verrier D., Mormede C., Kelley K.W., Dantzer R., Castanon N. (2005). Bacille Calmette-Guerin inoculation induces chronic activation of peripheral and brain indoleamine 2,3-dioxygenase in mice. J. Infect. Dis..

[168] Djoko K.Y., Ong C.L., Walker M.J., McEwan A.G. (2015). The Role of Copper and Zinc Toxicity in Innate Immune Defense against Bacterial Pathogens. J. Biol. Chem..

[169] White C., Lee J., Kambe T., Fritsche K., Petris M.J. (2009). A role for the ATP7A copper-transporting ATPase in macrophage bactericidal activity. J. Biol. Chem..

[170] Marcus S.A., Sidiropoulos S.W., Steinberg H., Talaat A.M. (2016). CsoR Is Essential for Maintaining Copper Homeostasis in *Mycobacterium tuberculosis*. PLoS ONE.

[171] Wolschendorf F., Ackart D., Shrestha T.B., Hascall-Dove L., Nolan S., Lamichhane G., Wang Y., Bossmann S.H., Basaraba R.J., Niederweis M. (2011). Copper resistance is essential for virulence of *Mycobacterium tuberculosis*. Proc. Natl. Acad. Sci. USA.

[172] Gold B., Deng H., Bryk R., Vargas D., Eliezer D., Roberts J., Jiang X., Nathan C. (2008). Identification of a copper-binding metallothionein in pathogenic mycobacteria. Nat. Chem. Biol..

[173] Bin B.H., Seo J., Kim S.T. (2018). Function, Structure, and Transport Aspects of ZIP and ZnT Zinc Transporters in Immune Cells. J. Immunol. Res..

[174] Botella H., Peyron P., Levillain F., Poincloux R., Poquet Y., Brandli I., Wang C., Tailleux L., Tilleul S., Charrière G.M. (2011). Mycobacterial p(1)-type ATPases mediate resistance to zinc poisoning in human macrophages. Cell Host Microbe.

[175] Abramovitch R.B., Rohde K.H., Hsu F.-F., Russell D.G. (2011). aprABC: A *Mycobacterium tuberculosis* complex-specific locus that modulates pH-driven adaptation to the macrophage phagosome. Mol. Microbiol..

[176] Baker J.J., Johnson B.K., Abramovitch R.B. (2014). Slow growth of *Mycobacterium tuberculosis* at acidic pH is regulated by phoPR and host-associated carbon sources. Mol. Microbiol..

[177] Chesne-Seck M.L., Barilone N., Boudou F., Gonzalo Asensio J., Kolattukudy P.E., Martín C., Cole S.T., Gicquel B., Gopaul D.N., Jackson M. (2008). A point mutation in the two-component regulator PhoP-PhoR accounts for the absence of polyketide-derived acyltrehaloses but not that of phthiocerol dimycocerosates in *Mycobacterium tuberculosis* H37Ra. J. Bacteriol..

[178] Frigui W., Bottai D., Majlessi L., Monot M., Josselin E., Brodin P., Garnier T., Gicquel B., Martin C., Leclerc C. (2008). Control of *M. tuberculosis* ESAT-6 secretion and specific T cell recognition by PhoP. PLoS Pathog..

[179] Walters S.B., Dubnau E., Kolesnikova I., Laval F., Daffe M., Smith I. (2006). The *Mycobacterium tuberculosis* PhoPR two-component system regulates genes essential for virulence and complex lipid biosynthesis. Mol. Microbiol..

[180] Gonzalo-Asensio J., Malaga W., Pawlik A., Astarie-Dequeker C., Passemar C., Moreau F., Laval F., Daffé M., Martin C., Brosch R. (2014). Evolutionary history of tuberculosis shaped by conserved mutations in the PhoPR virulence regulator. Proc. Natl. Acad. Sci. USA.

[181] Broset E., Martin C., Gonzalo-Asensio J. (2015). Evolutionary landscape of the *Mycobacterium tuberculosis* complex from the viewpoint of PhoPR: Implications for virulence regulation and application to vaccine development. mBio.

[182] Ates L.S., Dippenaar A., Sayes F., Pawlik A., Bouchier C., Ma L., Warren R.M., Sougakoff W., Majlessi L., van Heijst J.W.J. (2018). Unexpected Genomic and Phenotypic Diversity of *Mycobacterium africanum* Lineage 5 Affects Drug Resistance, Protein Secretion, and Immunogenicity. Genome Biol. Evol..

[183] Jackson M., Stadthagen G., Gicquel B. (2007). Long-chain multiple methyl-branched fatty acid-containing lipids of *Mycobacterium tuberculosis*: Biosynthesis, transport, regulation and biological activities. Tuberculosis.

[184] Rivero A., Márquez M., Santos J., Pinedo A., Sánchez M.A., Esteve A., Samper S., Martín C. (2001). High rate of tuberculosis reinfection during a nosocomial outbreak of multidrug-resistant tuberculosis caused by *Mycobacterium bovis* strain B. Clin. Infect. Dis..

[185] Soto C.Y., Menéndez M.C., Pérez E., Samper S., Gómez A.B., García M.J., Martín C. (2004). IS6110 mediates increased transcription of the phoP virulence gene in a multidrug-resistant clinical isolate responsible for tuberculosis outbreaks. J. Clin. Microbiol..

